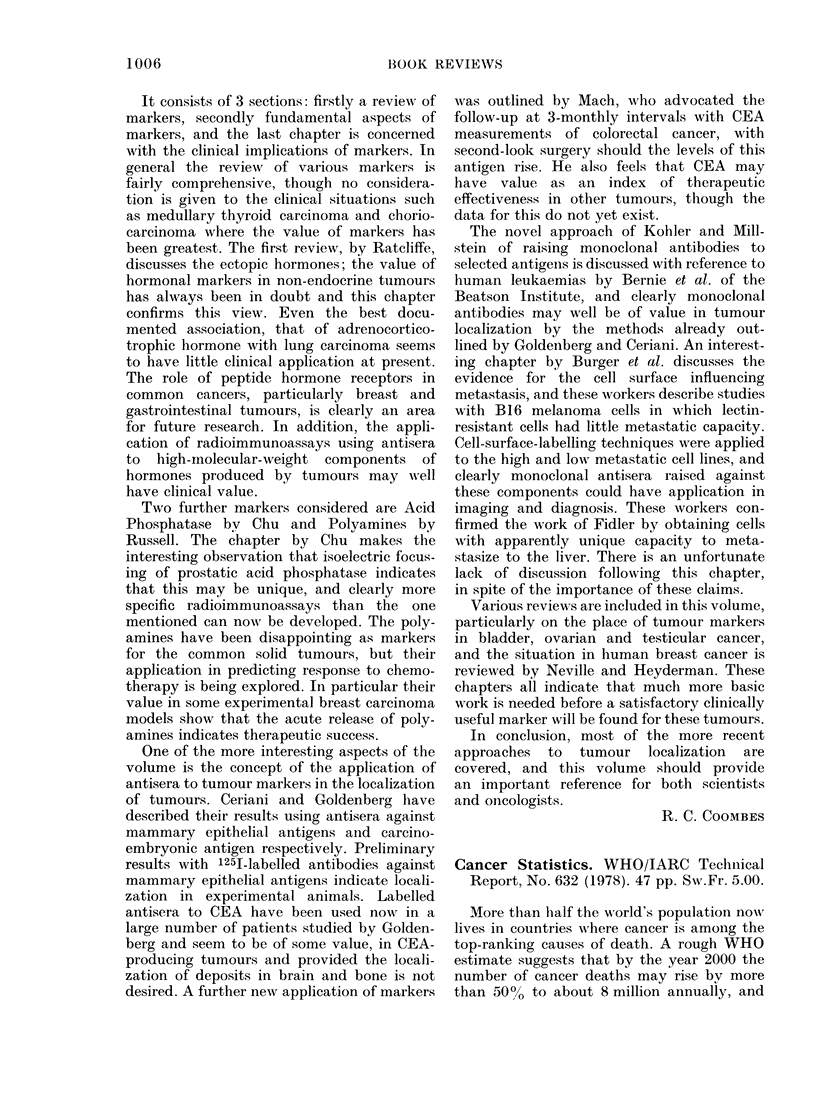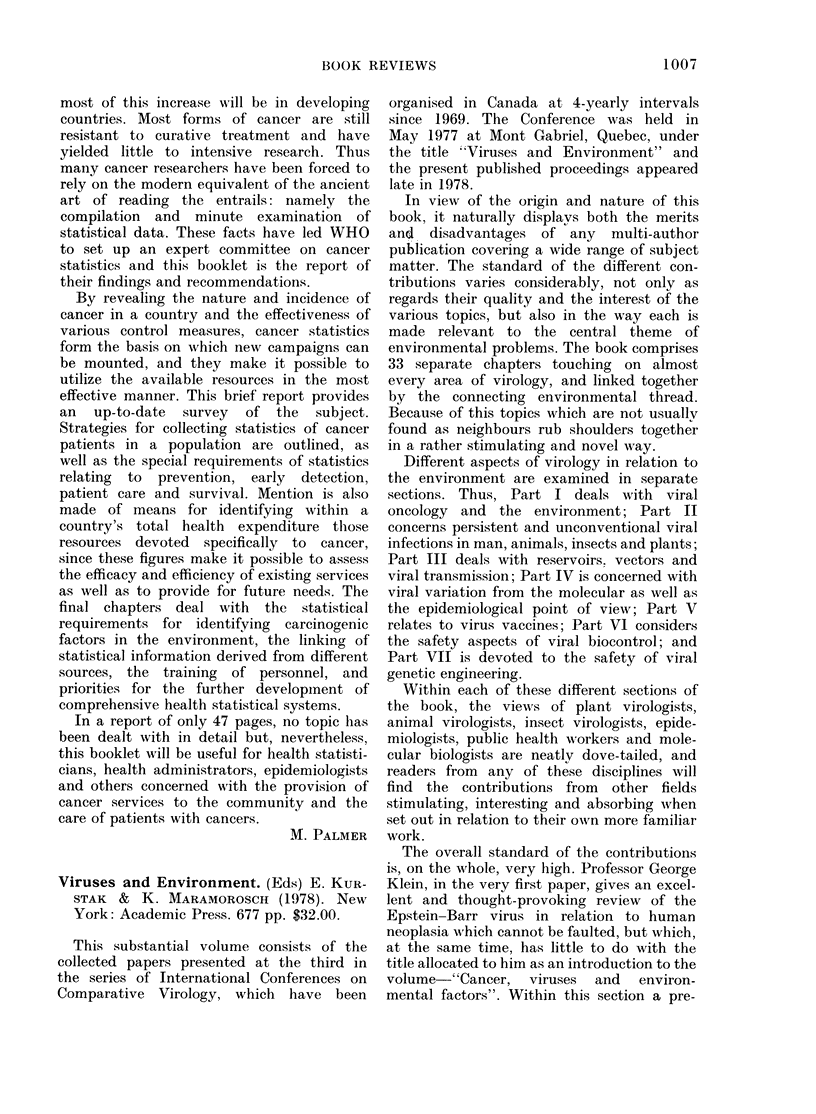# Cancer Statistics

**Published:** 1980-06

**Authors:** M. Palmer


**Cancer Statistics.** WHO/IARC Technical Report, No. 632 (1978). 47 pp. Sw.Fr. 5.00.

More than half the world's population now lives in countries where cancer is among the top-ranking causes of death. A rough WHO estimate suggests that by the year 2000 the number of cancer deaths may rise by more than 50% to about 8 million annually, and most of this increase will be in developing countries. Most forms of cancer are still resistant to curative treatment and have yielded little to intensive research. Thus many cancer researchers have been forced to rely on the modern equivalent of the ancient art of reading the entrails: namely the compilation and minute examination of statistical data. These facts have led WHO to set up an expert committee on cancer statistics and this booklet is the report of their findings and recommendations.

By revealing the nature and incidence of cancer in a country and the effectiveness of various control measures, cancer statistics form the basis on which new campaigns can be mounted, and they make it possible to utilize the available resources in the most effective manner. This brief report provides an up-to-date survey of the subject. Strategies for collecting statistics of cancer patients in a population are outlined, as well as the special requirements of statistics relating to prevention, early detection, patient care and survival. Mention is also made of means for identifying within a country's total health expenditure those resources devoted specifically to cancer, since these figures make it possible to assess the efficacy and efficiency of existing services as well as to provide for future needs. The final chapters deal with the statistical requirements for identifying carcinogenic factors in the environment, the linking of statistical information derived from different sources, the training of personnel, and priorities for the further development of comprehensive health statistical systems.

In a report of only 47 pages, no topic has been dealt with in detail but, nevertheless, this booklet will be useful for health statisticians, health administrators, epidemiologists and others concerned with the provision of cancer services to the community and the care of patients with cancers.

M. PALMER